# SPOC domain-containing protein Leaf inclination3 interacts with LIP1 to regulate rice leaf inclination through auxin signaling

**DOI:** 10.1371/journal.pgen.1007829

**Published:** 2018-11-29

**Authors:** Su-Hui Chen, Li-Juan Zhou, Ping Xu, Hong-Wei Xue

**Affiliations:** 1 National Key Laboratory of Plant Molecular Genetics, CAS Center for Excellence in Molecular Plant Sciences, Shanghai Institute of Plant Physiology and Ecology, Chinese Academy of Sciences, Shanghai, China; 2 University of Chinese Academy of Sciences, Beijing, China; 3 School of Agriculture and Biology, Shanghai Jiao Tong University, Shanghai, China; National University of Singapore and Temasek Life Sciences Laboratory, SINGAPORE

## Abstract

Leaf angle is an important agronomic trait and influences crop architecture and yield. Studies have demonstrated the roles of phytohormones, particularly auxin and brassinosteroids, and various factors in controlling leaf inclination. However, the underlying mechanism especially the upstream regulatory networks still need being clarified. Here we report the functional characterization of rice leaf inclination3 (LC3), a SPOC domain-containing transcription suppressor, in regulating leaf inclination through interacting with LIP1 (LC3-interacting protein 1), a HIT zinc finger domain-containing protein. LC3 deficiency results in increased leaf inclination and enhanced expressions of *OsIAA12* and *OsGH3*.*2*. Being consistent, transgenic plants with *OsIAA12* overexpression or deficiency of OsARF17 which interacts with OsIAA12 do present enlarged leaf inclination. LIP1 directly binds to promoter regions of *OsIAA12* and *OsGH3*.*2*, and interacts with LC3 to synergistically suppress auxin signaling. Our study demonstrate the distinct effects of IAA12-ARF17 interactions in leaf inclination regulation, and provide informative clues to elucidate the functional mechanism of SPOC domain-containing transcription suppressor and fine-controlled network of lamina joint development by LC3-regulated auxin homeostasis and auxin signaling through.

## Author summary

Leaf angle is a major trait of ideal architecture of crops that associates with photosynthetic efficiency and yields. Studies of the underlying mechanism will greatly help to improve the crop yield. Phytohormones especially auxin and brassinosteroids play crucial roles in regulating the leaf inclination, however, the upstream regulatory network is still unknown. Here, we functionally characterize a novel SPOC domain-containing protein LC3 (leaf inclination3) in lamina joint development through regulating auxin signaling. LC3 deficiency results in the excessive cell elongation at lamina joint adaxial side and hence the enlarged leaf angle. LC3 acts as a transcription suppressor through interacting with LIP1 (LC3-interacting protein 1, a HIT zinc finger domain-containing transcription factor), which directly binds to the promoters of auxin signaling and homeostasis related genes. Our studies provide new insights in the functional mechanism of SPOC domain-containing proteins and help to elucidate how auxin signaling is regulated during lamina joint development.

## Introduction

Rice is one of the most important crops in the world and breeding rice varieties with ideal architecture is a vital strategy for improvement of grain yields [1, 2]. Leaf is the main organ for photosynthesis and its development is crucial for the yield potential. Leaf inclination indicates the angle between leaf blade and culm [3], and studies have shown that erect leaf facilitates the penetration of sunlight and enhances the photosynthetic efficiency [4, 5], which is suitable for dense planting. Unbalanced development of collar cells at adaxial or abaxial sides, development of mechanical tissue and mechanical strength, formation of vascular bundle and cell wall compositions also have been pointed out to affect the leaf angle [2, 3, 6, 7].

Recent systemic analysis of the dynamic developmental processes of lamina joint through cytological observation showed that cell differentiation, division and elongation, cell wall thickening, and programmed cell death (PCD), are closely correlated with leaf angle and regulated by a complex network, consisting of various factors, especially protein kinases and hormones [8]. Indeed, studies by using mutants or transgenic approaches indicate that altered biosynthesis or signaling of brassinosteriods (BRs) lead to the change of leaf inclination, such as BR-deficient mutant *dwarf4-1* [9], *ebisudwarf* (*d2*) [10], *dwarf1* (*brd1*) [5], BR signaling mutant *d61-1*, *2* (weak mutant alleles of *OsBRI1*) [11], or rice plants with reduced expression of *OsBZR1* [12]. Similarly, plants with suppressed auxin signaling by overexpressing miR393a/b that suppress expression of receptor *OsTIR1* [13], or with reduced auxin levels including mutant *lc1* [6] or plants overexpressing *GH3* family members *OsGH3*.*2*, *OsGH3*.*5*, and *OsGH3*.*13* [7, 14, 15], present increased leaf inclination. It is noticed that BR stimulates while auxin suppresses the leaf inclination through regulating the cell division or elongation at adaxial side of lamina joint and auxin coordinates with BR to control the lamina joint development [6, 7, 16]. In addition, ethylene may participate in BR-induced leaf inclination [17] and repressed expression of a gibberellin signaling negative regulator, *SPINDLY*, leads to increased leaf angles [18].

Many transcription factors (TFs) are involved in leaf inclination regulation. *OsWRKY1* and MADS-box proteins OsMADS22, OsMADS55 and OsMADS47 negatively regulate leaf inclination [19–21]. Ectopic expression of LAX PANICLE (LAX), a basic helix-loop-helix TF, leads to increased bending of lamina joint [22]. Deficiency of OsLIGULELESS1 (OsLG1, a SBP domain-containing TF) results in defects in auricle, ligule, and lamina joint [23]. Recently, a genome-wide association study shows that rice TFs OsbHLH153, OsbHLH173 and OsbHLH174 involve in flag leaf angle regulation [24]. In addition, the Aux/IAA family members interact with AUXIN RESPONSE FACTOR (ARFs) to suppress auxin signaling [25] and regulate the leaf blades [26]. Overexpression of *OsIAA1*, *OsIAA4*, *OsARF19* lead to the increased leaf angle [7, 16, 27], while deficiency of OsARF11, an ortholog of *Arabidopsis ARF5*, results in reduced leaf angle [28]. However, the detailed mechanism, especially how Aux/IAA is regulated during lamina joint development and which distinct Aux/IAA-ARF interaction regulates leaf inclination is unknown yet.

By systemic analysis of a rice mutant with enlarged leaf angle, we showed that leaf inclination3 (LC3), a SPOC domain-containing protein that is speculated to facilitate protein-protein interactions in transcription repression complex [29], interacts with a HIT zinc finger domain-containing TF LIP1 (LC3-interacting protein 1) to suppress the auxin signaling and homeostasis genes, hence to regulate the cell elongation at adaxial side of lamina joint and thus leaf inclination. These results provide informative clues on the fine-controlled network regulating lamina joint development.

## Results

### *LC3* is expressed in lamina joint and knockout mutant *lc3* present enlarged leaf inclination due to the excessive cell elongation at adaxial side

Our previous studies by analyzing the global transcriptome of developing lamia joint showed that gene *leaf inclination3* (*LC3*, Os06g39480) is down-regulated from stage 2 to stage 6 during lamina joint development. Further analysis of the corresponding knockout mutant, *lc3*, revealed the obviously increased leaf angles under LC3 deficiency [8]. *LC3* encodes a novel SPOC domain-containing protein and the underlying functional mechanism is thus detailed studied.

**Fig 1:**
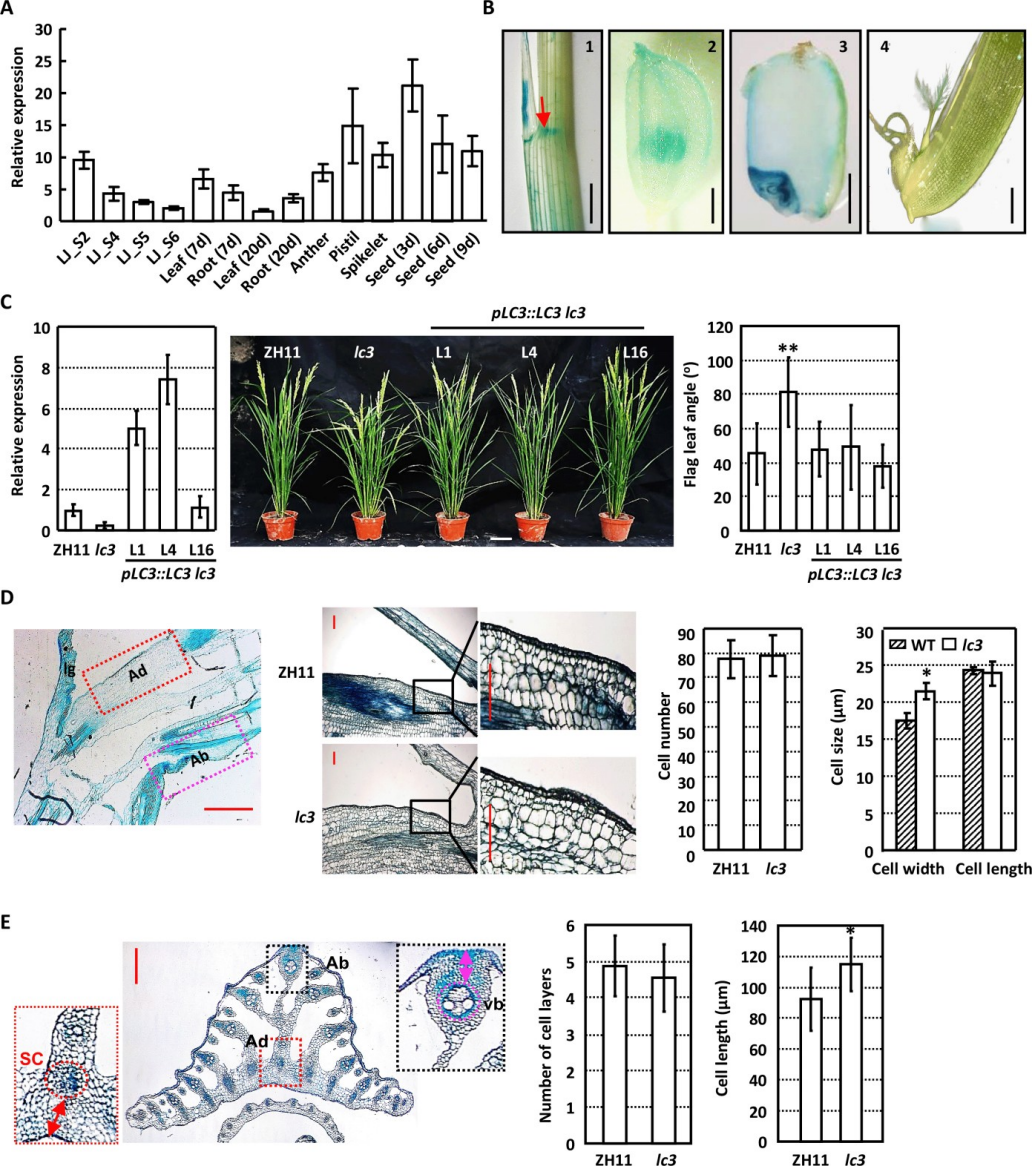
LC3 regulates rice leaf inclination. **(A)** qRT-PCR analysis reveals the *LC3* transcripts in various tissues, including lamina joint at different developmental stages (LJ_S2, S4, S5, S6 are four developmental stages of lamina joint according to Zhou et al., 2017), leaf, root and stem of 7- or 20-day-old plants, seed (3, 6, 9 d after fertilization), anther, pistil and spikelet. Transcription levels of *LC3* were normalized with that of *Actin* and *LC3* expression level in leaf at 20 days was set as 1.0. Analyses were repeated three times and data are shown as means ± SE (standard error). (B) Promoter-reporter gene (GUS) fusion studies reveal the *LC3* expression at lamina joint (1), glume and pollen (2), seed (3) and pistil (4). Representative images are shown. The red arrow indicates the adaxial side of collar. Bars = 2 mm (1–3) or 500 μm (4). (C)Phenotypic observations (middle, bar = 10 cm) and measurement (right) show that complemented *LC3* expression in *lc3* mutant (left) results in the recovered leaf inclination. Angles of rice flag leaf at 10 days after heading were measured and data are presented as means ± SD (standard deviation, n>30). Statistical analysis by using Student’s *t*-test reveals the significant differences compared to that of ZH11 (**, *p*<0.01). (D) Longitudinal section of the lamina joint of ZH11 flag leaves at 10 days after heading. ad, the adaxial region close to ligule; ab, the abaxial region away from ligule; lg, ligule (left, Bar = 400 μm). Morphology of longitudinal sections through the lamina joint of flag leaves of ZH11 and *lc3* plants at 10 days after heading are shown (middle, bar = 50 μm). The squared regions were magnified to highlight the differences. Cell number and size (cell width and length) of the second layer parenchyma cells at the adaxial side were calculated and statistically analyzed by Student’s t-test (right). Experiments were repeated three times and data are presented as means ± SD (n > 30; *, p<0.05). (E) Anatomy of the lamina joint of ZH11 flag leaves at 10 days after heading. ad, the region between the adaxial epidermis and sclerenchyma (sc) of the cross section; ab, the region between the abaxial epidermis and the abaxial central vascular bundle (vb) of the cross section (left, bar = 400 μm). The number of sclerenchyma cell layers (middle) and the length of ad (right) of ZH11 and *lc3* mutants were calculated and statistically analyzed by using Student’s t-test. Data are shown as means ± SD (n>30; *, *p*<0.05).

Analysis of the transcription pattern of *LC3* by quantitative real-time RT-PCR (qRT-PCR) confirms the reduced expression of *LC3* along with lamina joint development, while *LC3* is relatively highly expressed in pistil, spikelet and seeds at early stage (Fig 1A). Further promoter-reporter gene fusion analysis (a 3-kb promoter regions of *LC3* was fused to the β-glucuronidase gene) consistently show that *LC3* is transcribed at adaxial side of lamina joint, glume and pollen, pistil and seeds (Fig 1B). Based on the significantly decreased *LC3* expression in *lc3* [8], transgenic *lc3* plants with complemented expression of *LC3*, driven by its native promoter, was generated (Fig 1C, left panel). Phenotypic observation and measurement show the restored leaf inclination (Fig 1C), which confirms the role of LC3 in regulating leaf inclination.

To clarify the cytological change of *lc3* mutants, lamina joint paraffin section was conducted. Observations of the longitudinal sections show the increased cell width, while unaltered cell number and cell length of the second layer parenchyma cells at adaxial side of *lc3* lamina joint (Fig 1D). Further observations of transverse sections consistently show the increased cell length and unaltered cell layer numbers at adaxial side (Fig 1E), and no change of cell length and cell layers at abaxial side of *lc3* mutant lamina joint (S1 and S2 Figs). Overall, the excessive cell elongation at adaxial side of lamina joint results in the enlarged leaf angle of *lc3*.

### *LC3* deficiency results in the increased expression of *OsIAA12* and *OsGH3*.*2*

**Fig 2:**
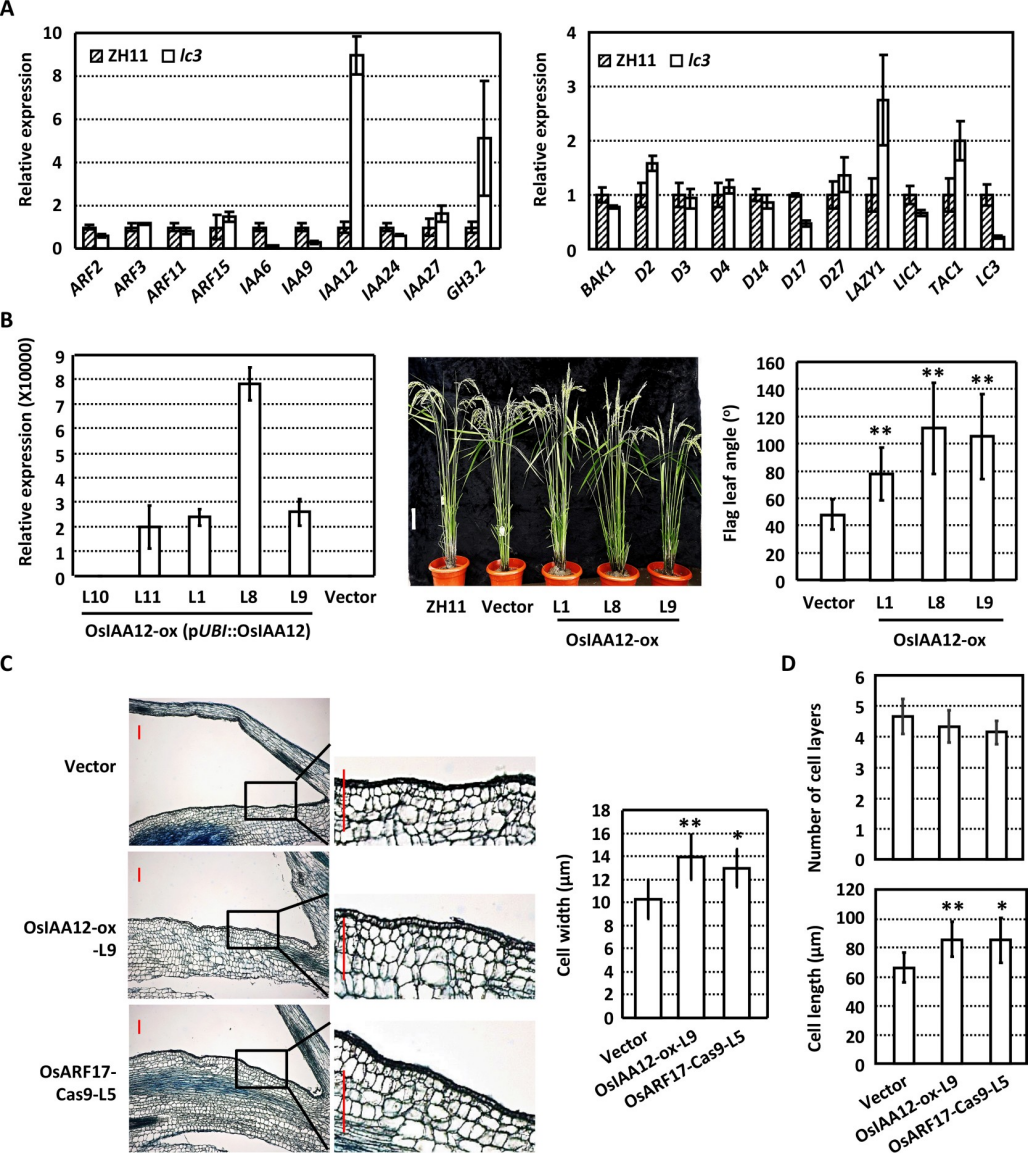
LC3 regulates auxin signaling and metabolism. **(A)** Expression levels of auxin and brassinosteroids (metabolism/synthesis, signaling) related genes that involve in leaf angle regulation in ZH11 and *lc3* mutant. Lamina joint of flag leaves at 10 days after heading were used for analysis. Transcription levels of examined genes were normalized with that of *Actin* and relative expressions are shown by comparing with the expression of corresponding genes in ZH11 (setting as “1.0”). Analyses were repeated three times and data are shown as means ± SE. (B) Phenotypic observations (middle, bar = 10 cm) and measurement (right) show that *OsIAA12* overexpression (left) results in the increased leaf angle. Transcription levels of *OsIAA12* were normalized with that of *Actin* and relative expressions were calculated by setting the *OsIAA12* expression in transgenic plants transformed with empty vector as “1.0”. Angles of rice flag leaf at 10 days after heading were measured. Experiments were biologically repeated three times and data are presented as means ± SE (n>30). Statistical analysis by using Student’s *t*-test reveals the significant differences (**, *p*<0.01). (C) Morphology of longitudinal sections through the lamina joint of flag leaves of rice transgenic plants containing empty vector, overexpressing *OsIAA12*, or deficiency of OsARF17 by Crispr/Cas9 (left, bar = 100 μm) at 10 days after heading. The boxed regions were magnified to highlight the differences. Cell width of the second layer of parenchyma cells at the adaxial side of the longitudinal sections were calculated (right). Data are shown as means ± SD (n>30) and statistically analyzed by using Student’s t-test (*, p<0.05; **, p<0.01). (D) The number of sclerenchyma cell layers (upper) and the length (bottom) of ad (shown in S3 Fig) were calculated and statistically analyzed by using Student’s t-test. Data are shown as means ± SD (n>30; *, *p*<0.05; **, *p*<0.01).

Auxin and brassinosteroids play crucial roles in regulating lamina joint development and thus leaf inclination. To investigate the functional mechanism of *LC3*, expression level of auxin and brassinosteroids signaling related genes and some reported genes regulating leaf angles were examined. qRT-PCR analysis reveals the decreased level of auxin signaling related genes *ARF2*, *IAA6*, *IAA9*, unaltered expressions of BR-related genes, increased expressions of *LAZY1* [30] and *TAC1* [31], and interestingly, dramatically increased levels of *OsIAA12* and *OsGH3*.*2* in *lc3* mutant (Fig 2A). Previous studies showed that overexpression of *OsGH3*.2 did result in the increased leaf angles [14], similar to *LC1* (OsGH3.1) overexpressing plants [6]. We thus focus on the effect of *OsIAA12* and relevant regulatory mechanism.

Transgenic rice plants overexpressing *OsIAA12* driven by a maize *ubiquitin* promoter were generated and analysis of the positive lines (Fig 2B, left panel) at 10 days after heading showed that *OsIAA12* overexpression indeed leads to the increased leaf inclination (Fig 2B). Analysis of the longitudinal sections of lamina joint reveals the increased cell width of the second layer parenchyma cells at adaxial side of *OsIAA12*-overexpressing lines (Fig 2C), which is same to that of *lc3*. Consistently, observations of the transverse sections of flag leaf lamina joint show that though there is no change in cell layer numbers in adaxial or abaxial regions (S3 and S4 Figs), increased length of adaxial cells was detected (Fig 2D). These results indicate that LC3 regulates lamina joint development possibly through *OsIAA12* and auxin signaling.

### OsIAA12 interacts with OsARF17 to regulate lamina joint development

**Fig 3:**
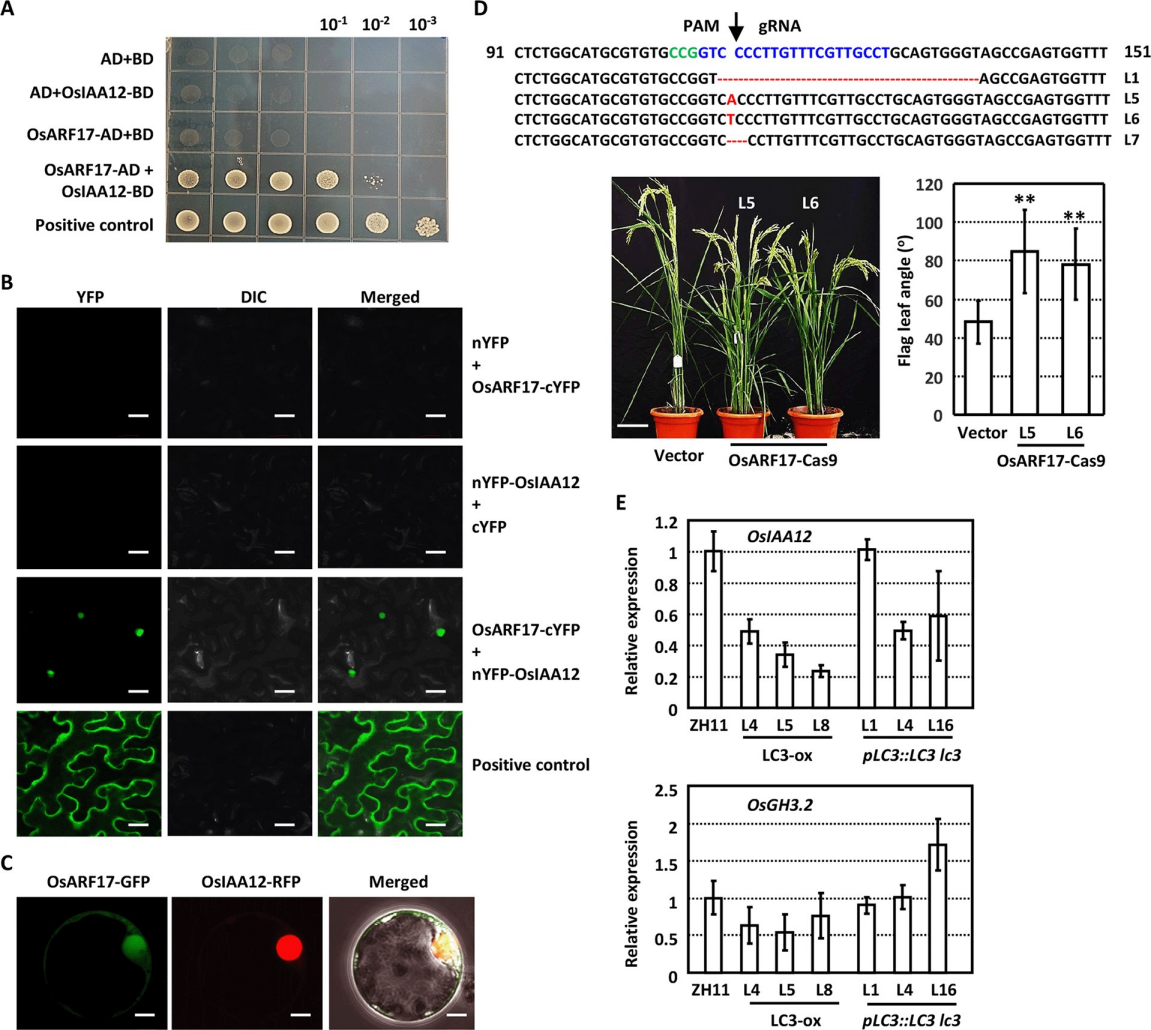
OsIAA12 interacts with OsARF17 to regulate leaf inclination. **(A)** Yeast-based two-hybrid analysis reveals the interaction between OsIAA12 and OsARF17. Yeast transformants were diluted 10^1^, 10^2^, and 10^3^ times and grown on synthetic dropout (-Leu/-Trp/-His/-Ade) plates for 4 days. (B) Split-YFP assays show the OsIAA12-OsARF17 interaction *in planta*. Fusion proteins OsARF17-cYFP and nYFP-OsIAA12 were expressed in *N*. *benthamiana* and fluorescences were observed. nYFP and cYFP were used as negative control. Bar = 30 μm. (C)OsIAA12 co-localizes with OsARF17 in cell nucleus. OsARF17-GFP and OsIAA12-RFP fusion protein were transiently co-expressed in rice protoplasts and representative images are shown. Bar = 5 μm. (D) Four homozygous knockout rice lines at *OsARF17* locus induced by Crispr/Cas9 were confirmed by sequencing. PAM (green) and gRNA (blue) sequences are highlighted. Mutations including deletions (red dots) and insertions (arrows). Phenotypic observation (bottom left, bar = 10 cm) and measurement of the leaf inclination at 10 days after heading (bottom right) show that *OsARF17*-Cas9 plants present enlarged leaf angle. Data are shown as means ± SD (n>30) and statistically analyzed by using Student’s t-test (**, p<0.01). (E)The expression of *OsIAA12* (upper) and *OsGH3*.*2* (bottom) in *LC3* overexpression and *lc3* plants with complemented *LC3* expression. Transcription levels were normalized with that of *Actin* and relative expressions were calculated by setting the expression level in ZH11 as “1.0”. Experiments were biologically repeated three times and data are presented as means ± SE.

AUX/IAA proteins interact with ARFs to suppress the auxin signaling, and which ARF cooperates with OsIAA12 to regulate the leaf inclination distinctly is studied. Previous studies on the IAAs-ARFs interacting networks indicated the interaction between OsIAA12 and OsARF17 [32], which was confirmed by the yeast two-hybrid assays (Fig 3A). Further analysis by Split-YFP assay through expressing N-terminal YFP fused OsIAA12 (nYFP-OsIAA12) and C-terminal YFP fused OsARF17 (OsARF17-cYFP) in tobacco leaf epidermal confirmed the OsIAA12-OsARF17 interaction in nucleus in *planta* (Fig 3B). Being consistent, transient expression of OsIAA12-RFP and OsARF17-GFP fusion proteins in rice protoplasts showed that OsIAA12 co-localizes with OsARF17 in nucleus (Fig 3C).

To confirm the role of OsIAA12-OsARF17 interaction in leaf inclination regulation, plants deficiency of OsARF17 were generated by Crispr/Cas9 approach (OsARF17-Cas9). Six independent transgenic lines were obtained and four of them were homozygous with either insertion or deletions at 5’ end of *OsARF17* (Fig 3D, upper panel). Phenotypic observations and measurement of T2 generations showed obviously enhanced flag leaf angles (Fig 3D, bottom panel). Analysis of paraffin section revealed similar cytological change as *lc3* mutant and transgenic plants overexpressing *OsIAA12* (Fig 2C and 2D; S3 and S4 Figs), suggesting that *LC3* regulates cell elongation at adaxial side and leaf inclination through suppressing the *OsIAA12* expression, which regulates OsARF17 effects by protein interaction. Examination of the transcriptions of *OsIAA12* and *OsGH3*.*2* showed the suppressed expression of *OsIAA12* and *OsGH3*.*2* under *LC3* overexpression and restored expression in *lc3* plants with complemented expression of *LC3* (Fig 3E), further confirming the regulation of *OsIAA12* and *OsGH3*.*2* by LC3. In addition, expression of *OsARF17* is unaltered under *OsIAA12* or *LC3* overexpression (S5 Fig), which is consistent with that Aux/IAA proteins repress ARF transcription factors via direct protein-protein interaction.

### LC3 interacts with LIP1 to suppress *OsIAA12* and *OsGH3*.*2* expressions

**Fig 4:**
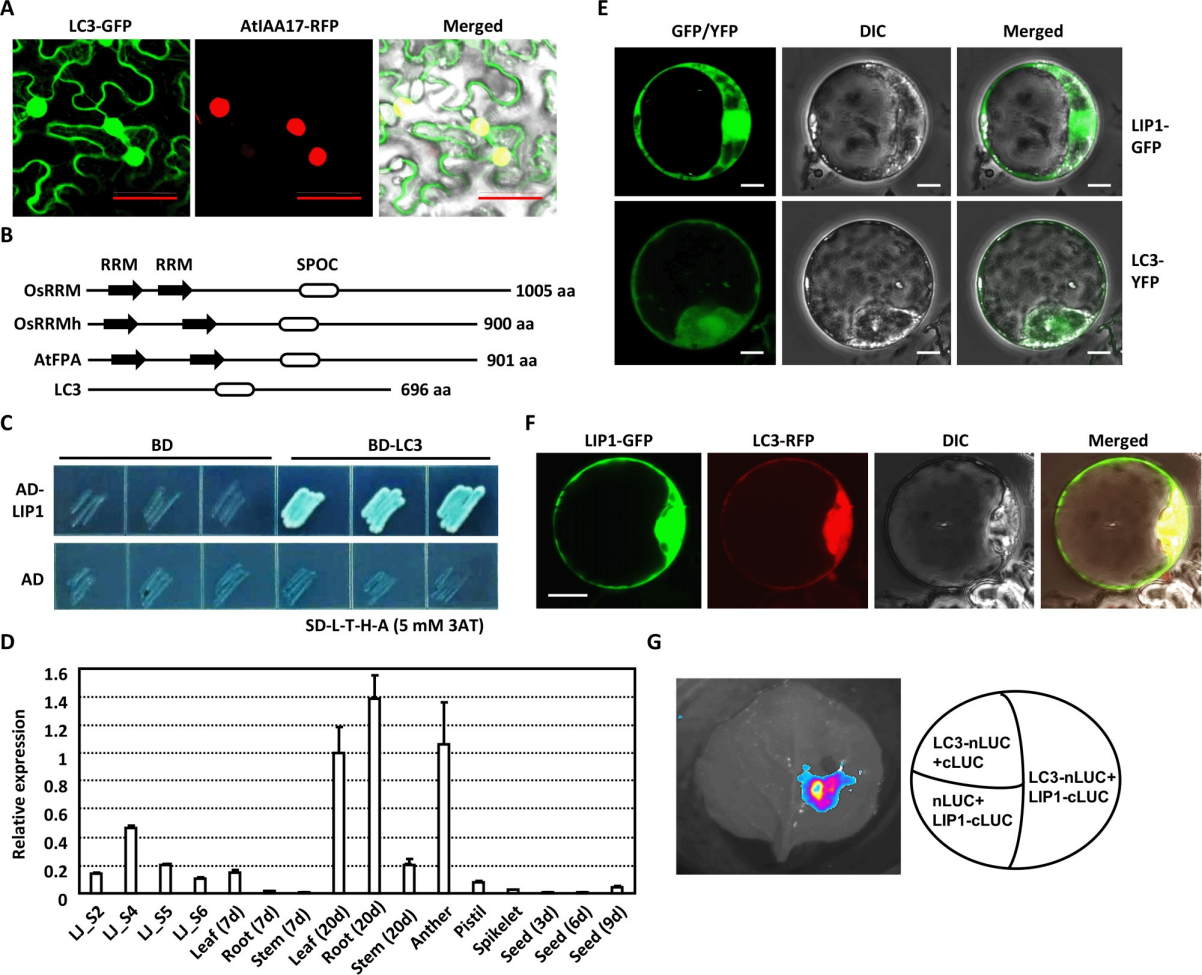
LC3 interacts with LIP1, a HIT zinc finger domain-containing protein. **(A)** Subcellular localization analysis reveals the ubiquitous LC3 distribution in nucleus, cytoplasm, and plasma membrane. LC3-GFP and nucleus-localized *Arabidopsis* AtIAA17-RFP fusion proteins were transiently co-expressed in *N*. *benthamiana* leaves and observed. Bar = 50 μm. (B) Structural organization of LC3 and comparison with representative proteins containing SPOC and conserved RRM (RNA recognition motif) domains in rice and *Arabidopsis*.(C) Yeast-based two-hybrid analysis reveals the LC3-LIP1 interaction. Yeast transformants were grown on synthetic dropout (-Leu/-Trp/-His/-Ade) plates plus X-α-Gal and 3-AT (3-amino-1, 2, 4-triazole, 5 mM) for 4 days. (D) qRT-PCR analysis reveals the *LIP1* transcript in various tissues. Transcription levels were normalized with that of *Actin* and *LIP1* expression level in leaf at 20 days was set as 1.0. Analyses were repeated three times and data are shown as means ± SE. (E)Subcellular localization analysis shows the localization of LC3 and LIP1. LC3-YFP and LIP1-GFP fusion proteins were transiently expressed in rice protoplasts. Bar = 5 μm. (F) LC3 co-localizes with LIP1 in cell nucleus and cytoplasm. LIP1-GFP and LC3-RFP fusion protein were transiently co-expressed in rice protoplasts, and representative images are shown. Bar = 10 μm. (G) Split-LUC (luciferase) assay shows the LC3-LIP1 interaction in *N*. *benthamiana* leaves. Fusion protein LC3-nLUC was co-transformed with LIP1-cLUC into tobacco leaves.

*LC3* encodes a SPOC-domain containing protein and localizes widely in cells (mainly in nucleus, Fig 4A). Previous reports showed that Spilt ends (Spen) protein family members compose an N-terminal RNA recognition motifs (RRM) domain and a conserved C-terminal SPOC domain [33, 34]. RRM domain regulates chromatin modification by recognizing and binding to DNA/RNAs specifically [34], while SPOC domain is proposed to facilitate protein-protein interactions in the transcription repression complex [29]. However, the underlying mechanism is unclear yet. In animals, Spen family members are reported to involve in neuron development, immune responses [29] and sex determination [35], which is less clarified in plants.

Phylogenetic analysis shows that there are three identified proteins close to LC3 (S6 Fig), including *Arabidopsis* FPA that controls flowering time [36]. OsRRMh and OsRRM, two rice homologues of AtFPA, that control flowering, fertility, and architecture [37, 38]. Protein structural analysis shows that compared to OsRRM, OsRRMh and AtFPA, the conserved RRM domain is absent in LC3 (Fig 4B), suggesting the distinct function of LC3. It is speculated that LC3 possibly interacts with other factors, which help to recognize DNA or RNA sequence and cooperate with LC3 to repress the transcription of downstream genes.

Yeast two-hybrid screening was thus conducted to isolate the candidate proteins that interact with LC3. Four proteins possibly interacting with LC3 were identified and designated as LIPs (LC3-interacting proteins). Further analysis confirmed the interaction between LC3 and LIP1, a HIT zinc finger domain-containing protein (Fig 4C). Transcription pattern analysis showed that *LIP1* presents similar expression pattern as *LC3* during lamina joint development (Fig 4D). Observation of fluorescence in rice protoplasts expressing LIP1-GFP/LC3-YFP revealed the similar localization of LIP1 and LC3 (Fig 4E). Furthermore, transient expression of LC3-RFP and LIP1-GFP fusion proteins in rice protoplasts showed that LC3 co-localizes with LIP1 both in nucleus and cytoplasm (Fig 4F). Split-Luciferase assay confirmed the interactions between LIP1 and LC3 *in vivo* (Fig 4G), indicating that LIP1 may coordinate with LC3 to regulate the leaf inclination.

**Fig 5:**
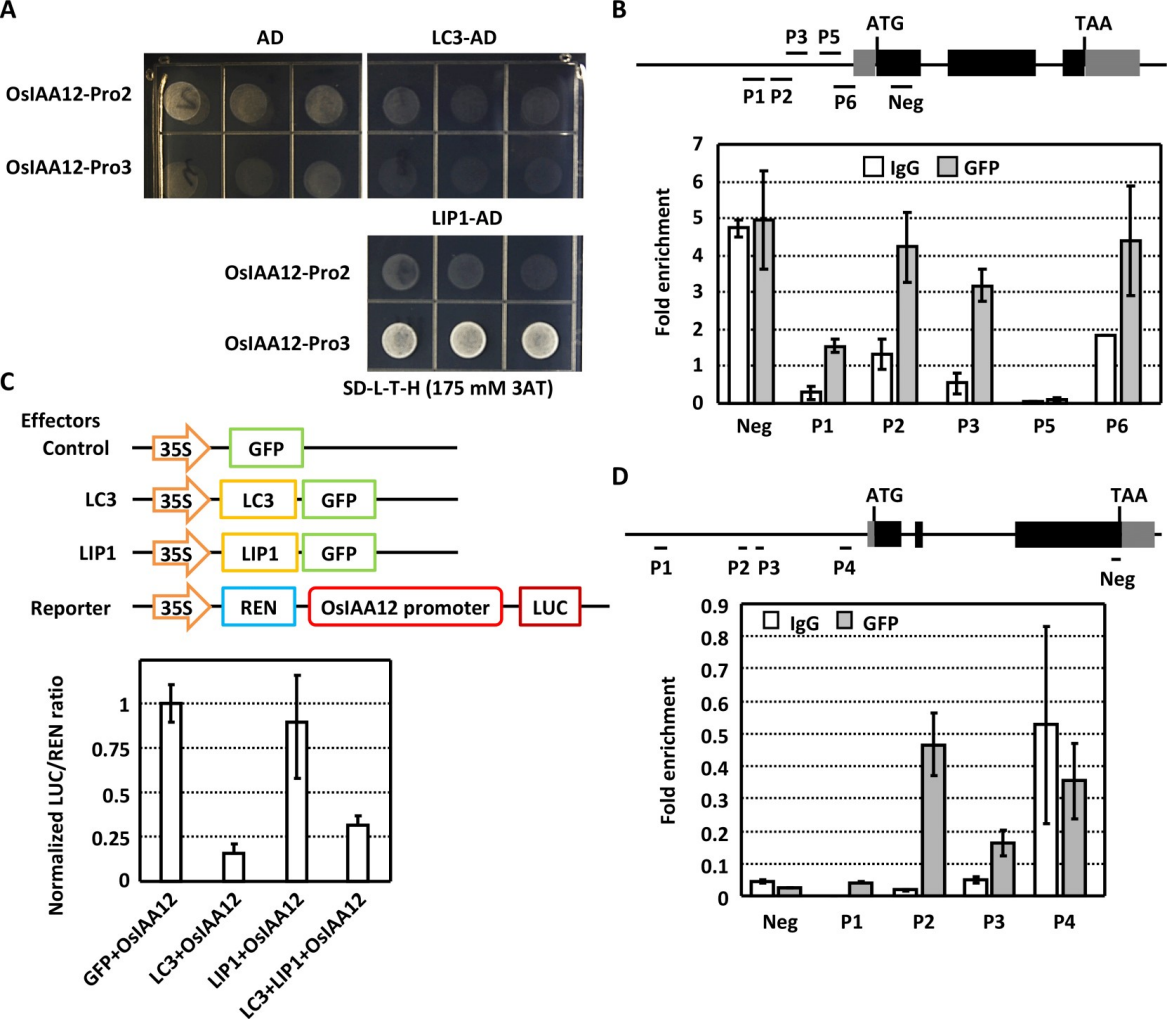
LIP1 directly binds to *OsIAA12* and *OsGH3*.*2* promoters. **(A)** Yeast one-hybrid analysis shows that LIP1, while LC3 does not, binds to the *OsIAA12* promoter region. Yeast transformants were grown on synthetic dropout (-Leu/-Trp/-His) plates with 3-AT (175 mM) for 3 days. Promoter regions Pro2 (-1709 to -915 bp) and Pro3 (-914 bp to 0 before the ATG) were subcloned into pHIS2 vector. AD vector was used as negative control.(B) ChIP-qPCR analysis demonstrates the direct binding of LC3 complex with *OsIAA12* promoter. Various DNA fragments (P1, P2, P3, P5, P6) of *OsIAA12* promoter were amplified by ChIP-qPCR using the indicated primer sets respectively. DNA fragment at coding region was amplified and used as a negative control. Experiments were repeated three times and results were normalized with the amounts of corresponding fragments in input. Data are shown as means ± SE (n>3). (C) Dual luciferase transcriptional activity assays show that LC3-LIP1 suppresses the *OsIAA12* transcription. Constructs for transient assays are schematically shown (upper). Both effector and reporter constructs were co-transformed into rice protoplasts. Relative activities of reporter activity (LUC) to the normalization standard (Renilla) were calculated by setting the relative ratio of LUC/REN in control as 1. Experiments were biologically repeated three times and data are presented as means ± SD. (D) ChIP-qPCR analysis demonstrates the direct binding of LC3 complex with *OsGH3*.*2* promoter. Several DNA fragments (P1-P4) of *OsGH3*.*2* promoters were amplified by ChIP-qPCR using the indicated primer sets respectively. Experiments were repeated three times and results were normalized with the amounts of corresponding fragments in input. Data are shown as means ± SE (n>3).

As LC3 lacks the RRM domain, it is hypothesized that LC3 may repress the downstream genes *OsIAA12* and *OsGH3*.*2* through interacting with LIP1, which recognize the binding sequences. Yeast one-hybrid analysis of *OsIAA12* promoter (fragments -1710 to 0 before ATG) showed that LIP1 binds to later region (-914 to 0 before ATG) but not the forward one, and LC3 binds to neither region (Fig 5A). In addition, by using 10-day-old transgenic seedlings expressing LC3-GFP, analysis of chosen five fragments in later region of *OsIAA12* promoter by quantitative chromatin immunoprecipitation (ChIP)-PCR indicated the enrichment of four DNA fragments (Fig 5B), confirming that LC3 binds to *OsIAA12* promoter through interacting with LIP1.

To further confirm the repression effect of LC3-LIP1 on downstream genes, two effector constructs carrying LC3 and LIP1 fusion GFP, were transiently expressed with a luciferase reporter (LUC) construct containing ~2.7-kb promoter of *OsIAA12* in rice protoplasts. Measurement showed that LUC expression was significantly reduced in the presence of LC3 or LC3-LIP1, and no differences in the presence of single LIP1 (Fig 5C), suggesting that LIP1 alone does not present inhibition effect, and LC3 and LIP1 cooperatively suppress the expressions of downstream genes. Similarly, ChIP-PCR assays of different DNA fragments in *OsGH3*.*2* promoter showed the enrichment of two DNA fragments (Fig 5D), indicating the binding of LC3 to *OsGH3*.*2* promoter as well. These results suggest that LIP1 orchestrates with LC3 to repress the *OsIAA12* and *OsGH3*.*2* expressions.

**Fig 6:**
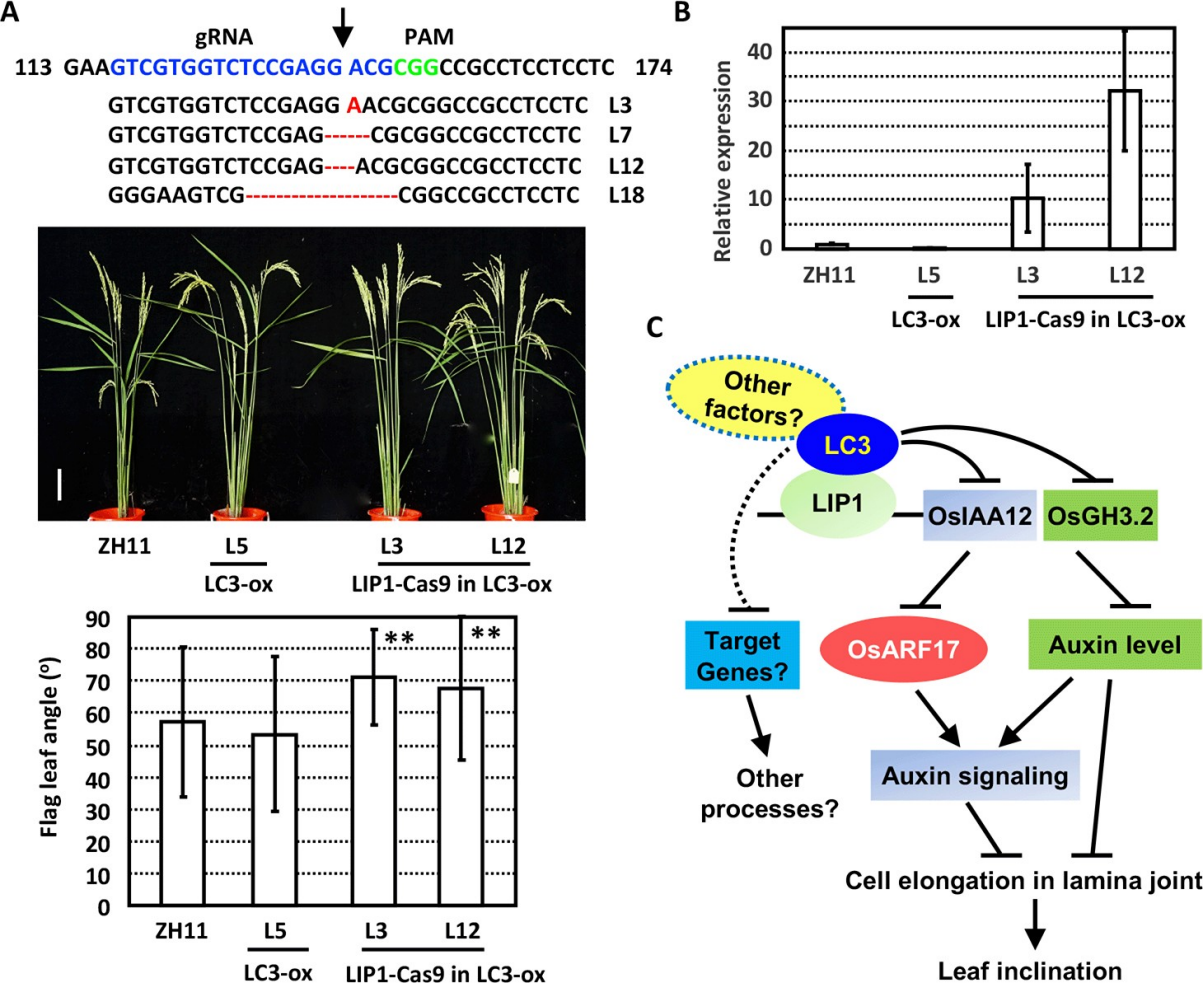
LC3-LIP1 controls leaf inclination through regulating auxin effects. (A) Four homozygous knockout rice lines at *LIP1* locus in *LC3* overexpression background induced by Crispr/Cas9 were confirmed by sequencing. PAM (green) and gRNA (blue) sequences are highlighted. Mutations including deletions (red dots) and insertions (arrows) (upper). Phenotypic observations (middle, bar = 10 cm) and measurement (bottom) of plants at 10 days after heading showed the enlarged leaf angles under LIP1 deficiency. Data are shown as means ± SD (n>30) and statistically analyzed by using Student’s t-test (**, p<0.01). (B) The expression of *OsIAA12* in ZH11, *LC3* overexpression (LC3-ox), and LIP1 deficiency in *LC3* overexpression background plants (LIP1-cas9 in LC3-ox). Transcription levels were normalized with that of *Actin* and relative expressions were calculated by setting the expression level in ZH11 as “1.0”. Experiments were biologically repeated three times and data are presented as means ± SE. (C) LC3-LIP1 module controls leaf inclination by regulating auxin effects. LC3 acts as a transcriptional suppressor and interacts with transcriptional factor LIP1 to repress the expressions of downstream genes. LIP1 directly binds to *OsIAA12* and *OsGH3*.*2* promoters. OsIAA12 interacts with OsARF17 to suppress the auxin signaling. Through suppressing the expressions of *OsIAA12* and *OsGH3*.*2*, LC3-LIP1 module promotes auxin signaling and hence suppresses the cell elongation at adaxial side of lamina joint, to maintain the normal leaf inclination. Deficiency of LC3 results in the increased expression of *OsIAA12* and *OsGH3*.*2* and hence reduced auxin level and signaling, leading to the enlarged cell width at adaxial side of lamina joint and increased leaf inclination.

There is no change of leaf angles under *LC3* overexpression (S7 Fig), indicating that LC3 functions to maintain the normal leaf inclination. To testify the function of LIP1 in lamina joint development, plants deficiency of LIP1 in background of *LC3* overexpression were generated by Crispr/Cas9 approach (LIP1-Cas9 in LC3-ox). Eighteen independent transgenic lines were obtained and four of them were homozygous with either insertion or deletions at 5’ end of *LIP1* (Fig 6A). Observation and analysis of the leaf inclination of plants in fields showed the increased leaf inclination under LIP1 deficiency (Fig 6A), indicating the crucial roles of LIP1 in mediating the LC3-LIP1 effects. In addition, the increased expression of *OsIAA12* under LIP1 deficiency (Fig 6B) further demonstrate that LIP1 and LC3 synergistically inhibit the transcription of *OsIAA12* expression.

## Discussion

SPOC-domain is speculated to facilitate protein-protein interactions in the transcription repression complex. Although SPOC domain-containing proteins are demonstrated to involve in regulation of various developmental processes, functions of them in plants are rarely reported. On the other hand, though auxin signaling/biosynthesis related genes are shown to affect the lamina joint development, the upstream regulation is still poorly understood. We functionally characterize a novel rice SPOC domain-containing protein leaf inclination 3 (LC3), whose deficiency (*lc3* mutant) presents enhanced leaf angle due to the excessive cell elongation at adaxial side of lamina joint, and demonstrate that LC3 controls leaf inclination by regulating auxin signaling through interacting with LIP1, a HIT zinc finger domain-containing transcriptional factor. It is therefore proposed that LC3 interacts with LIP1 to cooperatively suppress the expression levels of *OsIAA12* and *OsGH3*.*2*, resulting in the suppressed auxin signaling and homeostasis, to maintain the normal lamina joint development (Fig 6C). Our findings not only identify a novel factor regulating leaf inclination through auxin signaling and homoeostasis, but also reveal the function and underlying mechanism of a novel SPOC domain-containing protein.

Previous reports showed that RRM domain of SPOC domain-containing protein functions to recognize and bind to DNA/RNAs. Deficiency of RRM domain suggests that LC3 acts as a transcription repressor through interacting with other factors. Indeed, LIP1, a HIT zinc finger domain-containing TF, recognizes specific DNA sequence and forms a heterodimer with LC3 through interaction to suppress the transcription of downstream genes, especially *OsIAA12* and *OsGH3*.*2*. These illustrate the mechanism how LC3-LIP1 heterodimer represses the expression of auxin signaling and homeostasis related genes. In addition, it’s the first time to characterize the function and relevant mechanism of a SPOC-domain containing protein lacking RRM domain, which expands the knowledge on regulating the expression of downstream target genes in addition to RRM domain. A mouse Spen-like protein, MINT, binds to homeoprotein Msx2 to co-regulate osteocalcin [34, 39], and our results provide another example showing how SPOC-domain containing protein functions through interacting with a HIT zinc finger domain-containing protein, suggesting that SPOC-domain containing protein may interact with distinct TFs to suppress the transcription of specific genes, which provides novel insights for the functions of SPOC-domain containing proteins.

The underlying mechanism how LC3-LIP1 represses downstream target genes expression and whether there are other factors involving in the regulation, still need further investigations. In human, SHARP (SMRT/HDAC1-associated repressor protein), a spen protein, interacts with co-repressor SMRT (silencing mediator for retinoid and thyroid receptors) and NCoR (nuclear receptor corepressor), and these co-repressors repress transcription by recruiting a large complex containing histone deacetylase (HDAC) activity [29, 40]. In mice, Znhit1 binds to HDAC1 and suppresses *CDK6* expression by decreasing the histone H4 acetylation level in its promoter region [41]. Whether LC3 interacts with histone deacetylase or any other factors to repress the downstream gene transcription and hence regulates the distinct developmental processes needs further studies.

Plant phytohormone IAA plays crucial roles in lamina joint development, however, the upstream regulations of the key negative regulator Aux/IAAs during the process and which distinct IAA-ARF interaction is involved in lamina joint development control are unclear. We at first time demonstrate that a SPOC domain-containing protein LC3 regulates auxin signaling by directly suppress *OsIAA12* and auxin homeostasis through *OsGH3*.*2*. Aux/IAAs bind to ARFs to suppress its function [42] and as a multi-member family (there are 25 ARFs and 31 Aux/IAA proteins in rice), studies have revealed a complex interacting network of Aux/IAAs-ARFs that participate in regulation of various aspects of plant growth and development. Although it is known that each IAA protein can interact with different ARFs and each ARF protein can be suppressed by different IAAs to perform the diverse and specific functions [43], distinct functions of each interaction pair and how IAA-ARF interaction regulates leaf inclination remain to be elucidated. Our studies demonstrate the specific role of OsIAA12-OsARF17 interaction, which will help to illustrate the auxin effects in lamina joint development. Interestingly, other Aux/IAAs proteins (i.e. OsIAA20, OsIAA21, and OsIAA31) interact with OsARF17 besides OsIAA12, and OsIAA12 can also bind with OsARF21 [32], whether other Aux/IAAs-ARFs interactions regulate leaf inclination need further investigation. Further studies of the downstream genes of OsARF17 will expand the understanding of the detailed mechanism of OsIAA12-OsARF17 regulation in lamina joint development. In addition, the expression level of neither *LC3* nor *LIP1* is influenced by exogenous IAA treatment (S8 Fig), what kind of factors regulate LC3-LIP1 complex and hence lamina joint development will be interesting to be investigated.

*GH3* family members encode an indole-3-acetic acid-amido synthetase that conjugates free IAA to various amino acids [44, 45]. In addition to the regulation of *GH3* gene by ARFs, which is conserved among dicot and monocot plants [7], our results provide further understanding of *GH3* gene regulations by other regulators.

## Materials and methods

### Plant materials and growing conditions

Rice Zhonghua11 (ZH11, *Oryza sativa japonica* variety) plants, *lc3* mutant, and transgenic lines were grown in Shanghai and Lingshui (Hainan Province) under standard paddy conditions. Seedlings used to isolate protoplasts were grown in MS medium at 28°C with 12h-light/12-h dark cycle.

To analyze the expression pattern, lamina joints of flag leaf were collected from 60-, 65-, 70- or 80-day-old plants (stages 2, 4, 5, 6 according to definition by Zhou et al., 2017). Leaf, root, and stem were collected from 7- or 20-day-old seedlings. Seeds (3, 6, or 9 days after pollination), anther, pistil and spikelet were sampled.

For auxin treatment, 7-day-old seedlings were immersed in liquid 1/2 MS (Murashige and Skoog) medium containing IAA (indole-3-acetic acid, 10 μM) for 2 h and the collars were collected for qRT-PCR (quantitative real-time RT-PCR) analysis.

### Leaf angle measurement and cytological analysis

Leaf angle measurement and paraffin section were performed using plants 10 days after heading. Collected leaf lamina joints were photographed, and angle between sheath and leaf was measured by ImageJ program. At least 30 leaf angles of individual plants were measured.

For paraffin section analysis, leaf lamina joints were fixed in FAA solution (45% ethanol, 5% acetic acid, and 12.5% formaldehyde in water) for 24 h and dehydrated in a graded ethanol series and xylene-ethanol solution. Samples were embedded in paraffin (Sigma) for 1 day, then sections were cut (10 mm) and deparaffinized in xylene, hydrated through a graded ethanol series, and stained with Toluidine Blue. Extra stain was flushed and sections were dehydrated by a graded ethanol series again. Sections were microscopically observed and photographed, and cell number and cell size were calculated using ImageJ software.

### Vector construction and transformation

Entire *LC3* gene sequence including the 3-kb promoter region was amplified using primers LC3-3/LC3-4 and subcloned into pCAMBIA2300 for complementation study. Coding sequence of LC3 was amplified by primers LC3-13/LC3-14 and subcloned into pCAMBIA1300 driven by maize *ubiquitin* promoter for overexpression analysis. *LC3* promoter region was amplified using primers LC3-11/LC3-12 and subcloned into pCAMBIA1300+pBI101 vector [46] to drive the β-glucuronidase (GUS) gene. A binary vector pCAMBIA2300 carrying *OsIAA12* coding sequence amplified by primers IAA12-3/IAA12-4 and driven by *Zea mays ubiquitin* promoter was constructed for overexpressing *OsIAA12*. Transgenic rice with *OsARF17* mutation was generated by Crispr/Cas9 [47] with pOs-sgRNA using primers ARF17-1/ARF17-2. The gene editing construct for LIP1 deficiency via Crispr/Cas9 was designed using primers (LIP1-5/LIP1-6 and LIP1-7/LIP1-8) as previously described [48]. Confirmed constructs were transformed into rice by *Agrobacterium*-mediated transformation. Sequences of used primers were listed in S1 Table.

### Histochemical GUS staining

Various tissues were collected from the confirmed positive transgenic lines and incubated in substrate buffer (pH 7.0 NaH_2_PO_4,_ 0.1M; EDTA, 10 mM; K_4_Fe(CN)_6_, 0.5 mM; K_3_Fe(CN)_6_, 0.5 mM; 1% Trition X-100; 40 mg/mL X-Gluc). Examined samples were vacuumed and kept at 37°C overnight, then washed with 75% ethanol and observed.

### RNA extraction and qRT-PCR analysis

Total RNAs were extracted by Trizol reagent (Invitrogen) and used to synthesize cDNA through reverse transcription (Toyobo). qRT-PCR was conducted in a total volume of 20 μL containing 10 μL SYBR Premix Ex-Taq, 0.2 μL cDNA, primers (0.2 mM) and 8.3 μL double distilled water. Rice *Actin* gene was used as an internal control and transcription levels of *LC3*, *LIP1*, *OsIAA12*, *OsGH3*.*2*, *OsARF17* were examined using primers LC3-1/LC3-2, LIP1-9/LIP1-10, IAA12-1/IAA12-2, GH3.2-1/GH3.2–2, ARF17-9/ ARF17-10. Other primers used in qRT-PCR analysis were listed in S1 Table. All examinations were conducted with three biological and technological replicates.

### Yeast-two hybrid assays

Coding sequences of *LC3*, *LIP1*, *OsIAA12*, and *OsARF17* were amplified using primer LC3-5/LC3-6, LIP1-1/LIP1-2, IAA12-5/IAA12-6, and ARF17-3/ARF17-4 and subcloned into pGADT7 and pGBKT7 vectors respectively (Clontech). Confirmed constructs were co-transformed into yeast AH109 strain. Transformed yeast clones were diluted 10^1^, 10^2^, 10^3^ times, grown on synthetic dropout (SD) medium in the presence or absence of histidine with different concentrations of 3-amino-1, 2, 4-triazole (3-AT) according to the manufacturer’s instructions (Matchmaker user’s manual, Clontech, California), and observed after 4 days.

pGBKT7-LC3 vector was transformed into yeast strain Y2H Gold and used as bait in yeast-two hybrid screening analysis. The prey cDNAs derived from a rice cDNA library constructed from rice seedlings at different stages of ZH11 were transformed into yeast strain Y187. Mating bait and prey plasmid transformants were rotated at low speed for 20 h, then grown on synthetic dropout (SD) medium absence of histidine. Identified candidate prey cDNA was isolated from yeast cells and transformed into *Escherichia coli* for sequencing. Full cDNA sequence was amplified and cloned into pGADT7, which was co-transformed with pGBKT7-LC3 into yeast strain AH109 to verify the interaction.

### Subcellular localization studies

Coding sequence of *OsIAA12*, *OsARF17*, *LC3*, *LIP1* were amplified using primers IAA12-7/IAA12-8, ARF17-5/ARF17-6, LC3-9/LC3-10, LIP1-3/LIP1-4, LC3-15/LC3-16 and subcloned into pBI221-RFP [49] or pA7 vectors (C-terminus fusion with GFP or YFP) respectively. Resultant constructs expressing OsIAA12-RFP, OsARF17-GFP, LC3-YFP, LIP1-GFP, LC3-RFP fusion proteins were transformed into rice protoplasts and fluorescence was observed by confocal laser scanning microscope (FV10i, OLYMPUS) after 16 h.

### Split-YFP and Split-Luciferase assays

Coding sequences of *OsIAA12* and *OsARF17* were amplified using primers IAA12-9/IAA12-10, ARF17-7/ARF17-8 and subcloned into pCAMBIA1300S-YN and pCAMBIA1300S-YC vector by Infusion kit (Clontech) separately. Resultant constructs were transformed into *Agrobacterium tumefaciens* strain GV3101, which were used to infiltrate the leaves of 6-week-old tobacco plants. After infiltration for 48 h, YFP fluorescence was observed using a confocal laser scanning microscope (FV10i, OLYMPUS). Fusion proteins nYFP-OsHAL3 and cYFP-OsHAL3 were used as a positive control [50].

Coding sequences of *LC3* and *LIP1* were amplified and subcloned into the Gateway vector nLUC and cLUC respectively. After infiltration of tobacco leaves for 48 h, excess luciferin was sprayed on leaves and kept in dark for 10 min to eliminate the background fluorescence. Relative LUC activity was measured by a low light cooled CCD imaging apparatus at -70°C. Experiments were repeated three times for each assay.

### Yeast one-hybrid assays

Coding sequence of *LC3* and *LIP1* were amplified using primers LC3-7/LC3-8 and LIP1-1/LIP1-2, and subcloned into pGADT7 (Clontech). *OsIAA12* promoter regions Pro2 (-1709 to -915 bp before ATG) and Pro3 (-914 bp to 0 before ATG) were amplified using primers IAA12-11/IAA12-12, IAA12-13/IAA12-14 and subcloned into pHIS2 vector. Resultant constructs were transformed into yeast strain Y187. Yeast transformants were grown on synthetic dropout (-Leu/-Trp/-His) medium containing 175 mM 3-AT for 3 days and observed. Experiments were repeated three times.

### Quantitative ChIP-PCR analysis

ChIP-PCR assays were performed according to previous description [51]. Genomic DNAs extracted from 10-day-old transgenic seedling expressing LC3-GFP were digested into small pieces and crosslinked with formaldehyde. Resultant DNA fragments were sonicated to be ~200 bp in length. Chromatin immunoprecipitation were performed using anti-GFP antibody (ab290; Abcam), and Normal rabbit lgG (10500C; Invitrogen) was used as a negative control. Samples collected before immunoprecipitation were ‘input DNA’. Immunoprecipitated and input DNA were purified with PCR purification kit (Qiagen) and amplified using primers covering around 150-bp region of *OsIAA12* or *OsGH3*.*2* promoters by qPCR to examine the ChIP enrichment. Sequences of primers (IAA12-17 ~ IAA12-28, and GH3.2–3 ~ GH3.2–12) are listed in S1 Table. Fold-enrichment was calculated by normalizing the amount of a target DNA fragment against the respective input DNA samples. Experiments were repeated three times.

### Dual luciferase transcriptional activity assay

For effector constructs, coding regions of *LC3* and *LIP1* were amplified using primers LC3-9/LC3-10 and LIP1-3/LIP1-4 and subcloned into vector pA7 (C-terminus fusion with GFP). A ~2.7-kb DNA fragment of *OsIAA12* promoter was amplified by primers IAA12-15/IAA12-16 and subcloned into a modified pGreen0800 vector to generate the reporter construct. Effector and reporter constructs were co-transformed intro rice protoplasts. Dual-luciferase transcriptional activity assay was performed as previously described [52]. Experiments were biologically repeated three times.

### Transfection of rice protoplasts

Ten-day-old ZH11 seedlings were used to isolate protoplasts and 100 μL protoplasts suspension (containing ~2×10^5^ protoplasts) were transfected with plasmid (5–10 μg DNA) and 110 μL PEG solution. Transformation mixture was incubated in darkness for 15 min at 28°C, then diluted by 1 mL W5 solution (NaCl, 154 mM; CaCl_2_, 125 mM; D-Glucose, 5 mM; KCl, 5 mM; MES, 2 mM, pH 5.7) and centrifuged at 100 g for 2 min. Protoplasts were suspended in WI solution (Mannitol, 0.5 M; KCl, 20 mM, MES, 4 mM, pH 5.7) and transferred into multi well plates and incubated at 28°C for 16 h.

### Phylogenetic analyses

To construct a phylogenetic tree of SPOC-domain protein, homolog sequences in *A*. *thaliana*, *O*. *sativa* were obtained at the TAIR Web site (http://www.arabidopsis.org) and Rice Genome Annotation Project (http://rice.plantbiology.msu.edu). Alignment of available sequences was performed with CLUSTALX 1.83. The phylogenetic tree was constructed with MEGA 3 [53] using the neighbor-joining algorithm with 1001 bootstrap replicates.

### Accession numbers

All relevant data are within the paper and its Supporting Information files except for the sequence data which is available from the rice genome database RICEGE (http://signal.salk.edu/cgi-bin/RiceGE) or GenBank databases (https://www.ncbi.nlm.nih.gov/genbank/) under the following accession numbers: LC3 (Os06g0595900), LIP1 (Os10g0520700), OsIAA12 (Os03g0633800), OsARF17 (Os06g0677800).

## Supporting information

S1 Fig *lc3* plant presents enlarged cell width at adaxial side of the flag leaf collar. The cross-sections of the adaxial region of ZH11 and *lc3* flag leaf collars at 10 days after heading were shown. Bar = 50 μm.
(PDF)

S2 Fig *lc3* plant presents no change in abaxial region of flag leaf collar. A. The cross-sections of the abaxial region of the flag leaf collar of ZH11 and *lc3* plants. Bar = 50 μm.
B. The number of cell layers (left) and cells length (right) of abaxial region of collar (shown in S2A) were calculated and statistical analysis by using Student’s t-test revealed no differences. Data are shown as means ± SD (n>30).
(PDF)

S3 Fig Enlarged cell width at adaxial side of the flag leaf collar of rice plants overexpressing *OsIAA12* or deficiency of OsARF17 by Crispr/Cas9. The cross-sections of the adaxial region of the flag leaf collar of rice plants overexpressing *OsIAA12* or deficiency of OsARF17 by Crispr/Cas9 at 10 days after heading were shown. Bar = 50 μm.
(PDF)

S4 Fig Rice plants overexpressing *OsIAA12* or deficiency of OsARF17 present no change in abaxial region of flag leaf collar. A. The cross-sections of the abaxial region of the flag leaf collar of rice plants overexpressing *OsIAA12* or deficiency of OsARF17 by Crispr/Cas9 at 10 days after heading. Bar = 50 μm.
B. The number of cell layers (left) and cell length (right) of abaxial region of collar (shown in S4A) were calculated and statistical analysis by using Student’s t-test revealed no differences. Data are shown as means ± SD (n>30).
(PDF)

S5 Fig The expression of *OsARF17* in *lc3* with complemented *LC3* expression, *LC3* overexpression, *lc3* and *OsIAA12* overexpression plants. Transcription levels of *OsARF17* were normalized with that of *Actin* and relative expressions were calculated by setting the *OsARF17* expression level in ZH11 as “1.0”. Experiments were biologically repeated three times and data are presented as means ± SE (n>3).
(PDF)

S6 Fig Phylogenetic tree of the SPOC domain-containing proteins in rice and *Arabidopsis*. Protein sequences were obtained from NCBI, and characterized OsRRM, OsRRMh, and AtFPA were indicated. Phylogenetic tree was generated using MEGA3 software.
(PDF)

S7 Fig *LC3* overexpression does not change rice leaf inclination. A. qRT-PCR analysis confirms the enhanced *LC3* expression in ZH11 plants transformed with pUBI::LC3. Lamina joint of flag leaf at 10 days after heading was analyzed. Transcript levels of *LC3* were normalized with that of *Actin* and *LC3* expression in ZH11 flag leaf was set as 1.0. Analyses were repeated three times and data are shown as means ± SD (n>3).
B. Phenotypic observations (upper, bar = 10 cm) and measurement (bottom) show that enhanced *LC3* expression does not alter the leaf inclination. Angles of rice flag leaf at 10 days after heading were measured and statistically analyzed by using Student’s *t*-test. Experiments were repeated three times and data are presented as means ± SD (n>30; *, *p*<0.05).
(PDF)

S8 Fig Expression of *LC3* and *LIP1* is not regulated by auxin treatment. Seven-day-old seedlings grown in normal culture solution were treated with 10 μM indole-3-acetic acid (IAA) for 2 h. Transcription levels of *LC3* and *LIP1* were normalized with that of *Actin* and relative expressions were calculated by setting the expression of corresponding genes in Mock as “1.0”. Experiments were biologically repeated three times and data are presented as means ± SE (n>3).
(PDF)

S1 Table Primers used for qRT-PCR and plasmid construction. Restriction endonuclease sites are underlined.
(PDF)
