# Supplementary material for: SPOC domain-containing protein Leaf inclination3 interacts with LIP1 to regulate rice leaf inclination through auxin signaling
Source: PLoS Genet. 2018 Nov 29;14(11):e1007829. doi: 10.1371/journal.pgen.1007829 (PMC6289470; doi:10.1371/journal.pgen.1007829)
Supplement: S1 Fig — The cross-sections of the adaxial region of ZH11 and lc3 flag leaf collars at 10 days after heading were shown. Bar = 50 μm. (PDF) [file pgen.1007829.s001.pdf]

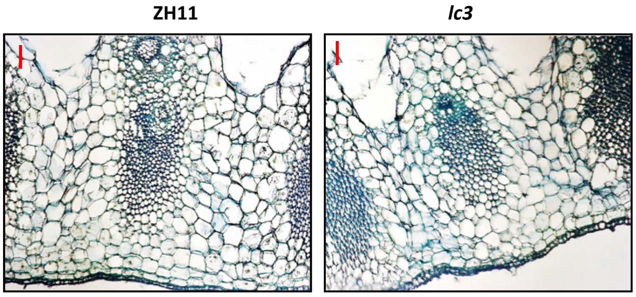

S1 Fig. *lc3* plant presents enlarged cell width at adaxial side of the flag leaf collar. The cross-sections of the adaxial region of ZH11 and *lc3* flag leaf collars at 10 days after heading were shown. Bar=50 μm.
