# Supplementary material for: SPOC domain-containing protein Leaf inclination3 interacts with LIP1 to regulate rice leaf inclination through auxin signaling
Source: PLoS Genet. 2018 Nov 29;14(11):e1007829. doi: 10.1371/journal.pgen.1007829 (PMC6289470; doi:10.1371/journal.pgen.1007829)
Supplement: S2 Fig — A. The cross-sections of the abaxial region of the flag leaf collar of ZH11 and lc3 plants. Bar = 50 μm. B. The number of cell layers (left) and cells length (right) of abaxial region of collar (shown in S2A) were calculated and statistical analysis by using Student’s t-test revealed no differences. Data are shown as means ± SD (n>30). (PDF) [file pgen.1007829.s002.pdf]

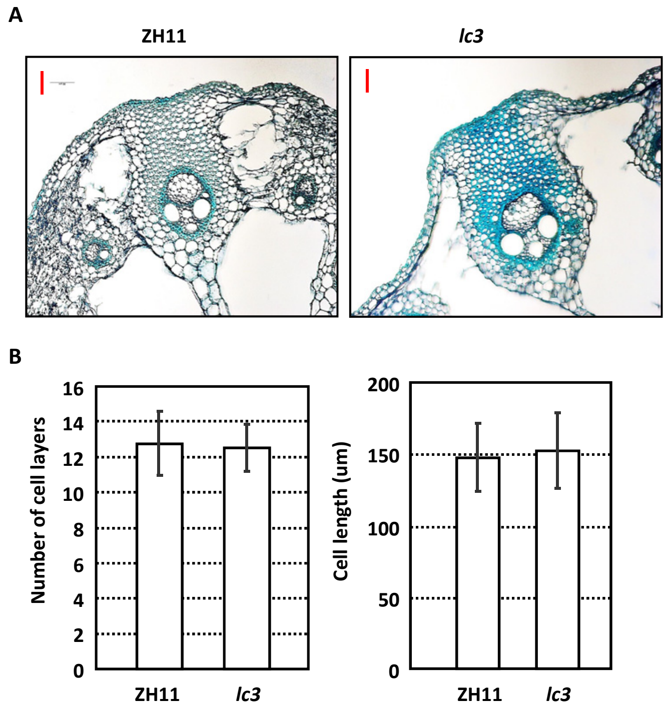

S2 Fig. *lc3* plant presents no change in abaxial region of flag leaf collar.

A. The cross-sections of the abaxial region of the flag leaf collar of ZH11 and *lc3* plants. Bar=50 μm.

B. The number of cell layers (left) and cells length (right) of abaxial region of collar (shown in S2A) were calculated and statistical analysis by using Student's t-test revealed no differences. Data are shown as means ± SD (n>30).
