# Supplementary material for: SPOC domain-containing protein Leaf inclination3 interacts with LIP1 to regulate rice leaf inclination through auxin signaling
Source: PLoS Genet. 2018 Nov 29;14(11):e1007829. doi: 10.1371/journal.pgen.1007829 (PMC6289470; doi:10.1371/journal.pgen.1007829)
Supplement: S3 Fig — The cross-sections of the adaxial region of the flag leaf collar of rice plants overexpressing OsIAA12 or deficiency of OsARF17 by Crispr/Cas9 at 10 days after heading were shown. Bar = 50 μm. (PDF) [file pgen.1007829.s003.pdf]

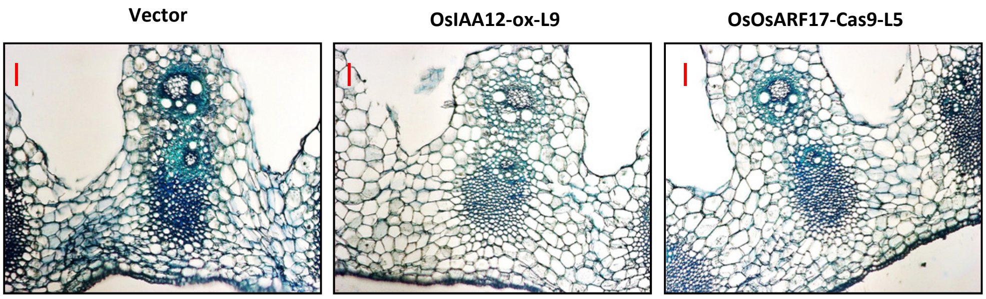

S3 Fig. Enlarged cell width at adaxial side of the flag leaf collar of rice plants overexpressing *OsIAA12* or deficiency of OsARF17 by Crispr/Cas9. The cross-sections of the adaxial region of the flag leaf collar of rice plants overexpressing *OsIAA12* or deficiency of OsARF17 by Crispr/Cas9 at 10 days after heading were shown. Bar=50 µm.
