# Supplementary material for: SPOC domain-containing protein Leaf inclination3 interacts with LIP1 to regulate rice leaf inclination through auxin signaling
Source: PLoS Genet. 2018 Nov 29;14(11):e1007829. doi: 10.1371/journal.pgen.1007829 (PMC6289470; doi:10.1371/journal.pgen.1007829)
Supplement: S5 Fig — Transcription levels of OsARF17 were normalized with that of Actin and relative expressions were calculated by setting the OsARF17 expression level in ZH11 as “1.0”. Experiments were biologically repeated three times and data are presented as means ± SE (n>3). (PDF) [file pgen.1007829.s005.pdf]

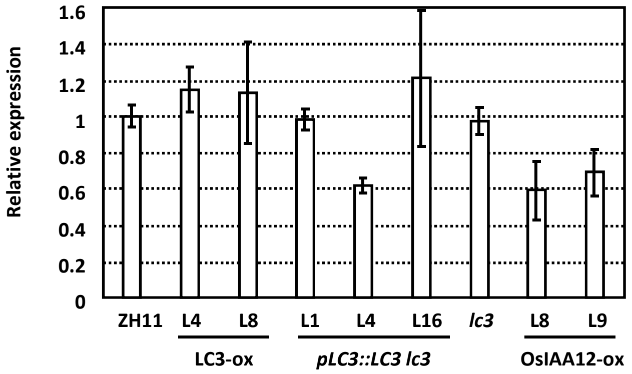

S5 Fig. The expression of *OsARF17* in *lc3* with complemented *LC3* expression, *LC3* overexpression, *lc3* and *OsIAA12* overexpression plants. Transcription levels of *OsARF17* were normalized with that of *Actin* and relative expressions were calculated by setting the *OsARF17* expression level in ZH11 as "1.0". Experiments were biologically repeated three times and data are presented as means ± SE (n>3).
