# Supplementary material for: SPOC domain-containing protein Leaf inclination3 interacts with LIP1 to regulate rice leaf inclination through auxin signaling
Source: PLoS Genet. 2018 Nov 29;14(11):e1007829. doi: 10.1371/journal.pgen.1007829 (PMC6289470; doi:10.1371/journal.pgen.1007829)
Supplement: S6 Fig — Protein sequences were obtained from NCBI, and characterized OsRRM, OsRRMh, and AtFPA were indicated. Phylogenetic tree was generated using MEGA3 software. (PDF) [file pgen.1007829.s006.pdf]

Chen et al., S6 Fig

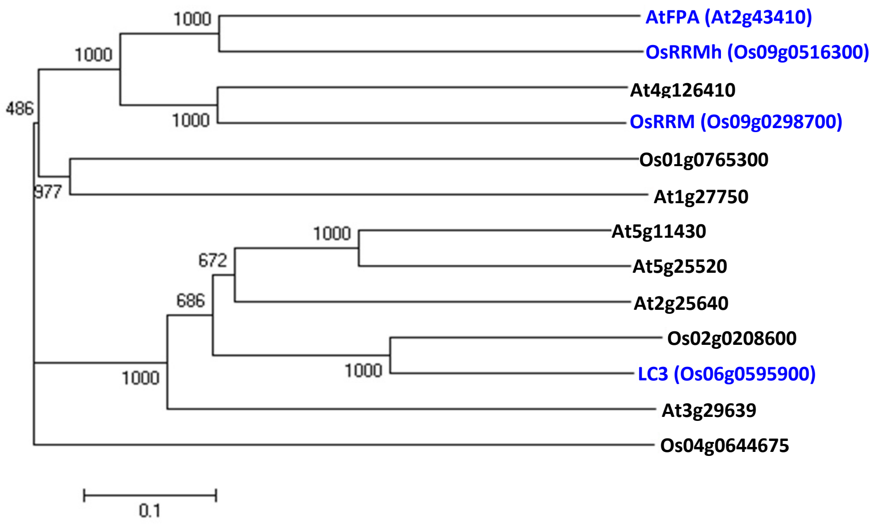


**S6 Fig. Phylogenetic tree of the SPOC domain-containing proteins in rice and *Arabidopsis*.** Protein sequences were obtained from NCBI，and characterized OsRRM, OsRRMh, and AtFPA were indicated. Phylogenetic tree was generated using MEGA3 software.
