# Supplementary material for: SPOC domain-containing protein Leaf inclination3 interacts with LIP1 to regulate rice leaf inclination through auxin signaling
Source: PLoS Genet. 2018 Nov 29;14(11):e1007829. doi: 10.1371/journal.pgen.1007829 (PMC6289470; doi:10.1371/journal.pgen.1007829)
Supplement: S7 Fig — A. qRT-PCR analysis confirms the enhanced LC3 expression in ZH11 plants transformed with pUBI::LC3. Lamina joint of flag leaf at 10 days after heading was analyzed. Transcript levels of LC3 were normalized with that of Actin and LC3 expression in ZH11 flag leaf was set as 1.0. Analyses were repeated three times and data are shown as means ± SD (n>3). B. Phenotypic observations (upper, bar = 10 cm) and measurement (bottom) show that enhanced LC3 expression does not alter the leaf inclination. Angles of rice flag leaf at 10 days after heading were measured and statistically analyzed by using Student’s t-test. Experiments were repeated three times and data are presented as means ± SD (n>30; *, p<0.05). (PDF) [file pgen.1007829.s007.pdf]

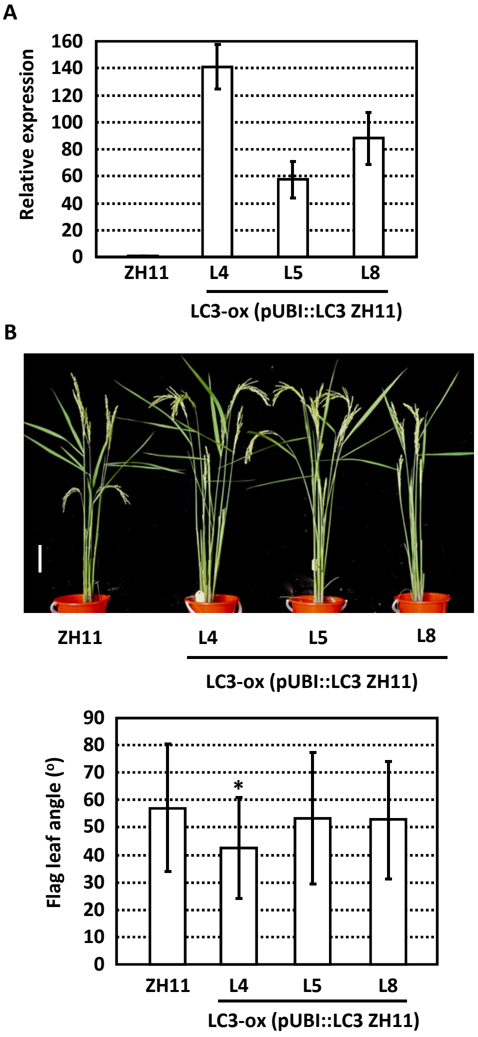

S7 Fig. *LC3* overexpression does not change rice leaf inclination.

A. qRT-PCR analysis confirms the enhanced *LC3* expression in ZH11 plants transformed with pUBI::LC3. Lamina joint of flag leaf at 10 days after heading was analyzed. Transcript levels of *LC3* were normalized with that of *Actin* and *LC3* expression in ZH11 flag leaf was set as 1.0. Analyses were repeated three times and data are shown as means ± SD (n>3).

B. Phenotypic observations (upper, bar=10 cm) and measurement (bottom) show that enhanced *LC3* expression does not alter the leaf inclination. Angles of rice flag leaf at 10 days after heading were measured and statistically analyzed by using Student's *t*-test. Experiments were repeated three times and data are presented as means ± SD (n>30; *, p<0.05).
