# Supplementary material for: SPOC domain-containing protein Leaf inclination3 interacts with LIP1 to regulate rice leaf inclination through auxin signaling
Source: PLoS Genet. 2018 Nov 29;14(11):e1007829. doi: 10.1371/journal.pgen.1007829 (PMC6289470; doi:10.1371/journal.pgen.1007829)
Supplement: S8 Fig — Seven-day-old seedlings grown in normal culture solution were treated with 10 μM indole-3-acetic acid (IAA) for 2 h. Transcription levels of LC3 and LIP1 were normalized with that of Actin and relative expressions were calculated by setting the expression of corresponding genes in Mock as “1.0”. Experiments were biologically repeated three times and data are presented as means ± SE (n>3). (PDF) [file pgen.1007829.s008.pdf]

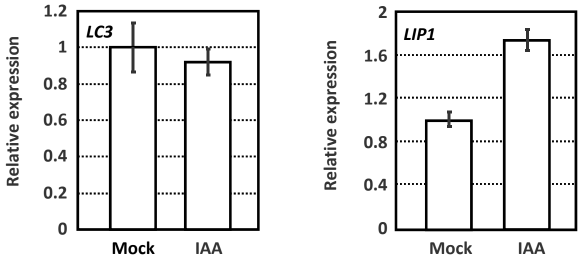

S8 Fig. Expression of *LC3* and *LIP1* is not regulated by auxin treatment. Seven-day-old seedlings grown in normal culture solution were treated with 10 μM indole-3-acetic acid (IAA) for 2 h. Transcription levels of *LC3* and *LIP1* were normalized with that of *Actin* and relative expressions were calculated by setting the expression of corresponding genes in Mock as "1.0". Experiments were biologically repeated three times and data are presented as means ± SE (n>3).
