# Supplementary material for: SPOC domain-containing protein Leaf inclination3 interacts with LIP1 to regulate rice leaf inclination through auxin signaling
Source: PLoS Genet. 2018 Nov 29;14(11):e1007829. doi: 10.1371/journal.pgen.1007829 (PMC6289470; doi:10.1371/journal.pgen.1007829)
Supplement: S1 Table — Restriction endonuclease sites are underlined. (PDF) [file pgen.1007829.s009.pdf]

S1 Table. Primers used for qRT-PCR and plasmid construction. Restriction endonuclease sites are underlined

Primers	Sequences (5'-3')	Restriction enzymes
Actin-1	CCTTCAACACCCCTGCTATG	
Actin-2	TGAGTAACCACGCTCCGTCA	
LC3-1	GGTGAAAAGACATCCACAAACGA	
LC3-2	TATGAGTCAATGGTCCGCAAGA	
LC3-3	CCCAAGCTTACGGGTGATGGGCTCTTGGAG	*Hind*III
LC3-4	CCCAAGCTTCCAGCAACTACCTAAGCGG	*Hind*III
LC3-5	GGAATTCCATATGACTACTGAAGAACTCGCTTCAA	*Nde* I
LC3-6	TCCCCCGGGCTACTGTAGGTTCCAAGGCATGG	*Sma* I
LC3-7	CCCATATGATGACTACTGAAGAACTCGC	*Nde* I
LC3-8	CGAGCTCGCTGTAGGTTCCAAGGCATGG	*Sac* I
LC3-9	TGGTCGACGTACTAGTATGACTACTGAAGAACTCGC	*Spe* I
LC3-10	GCTCACCATCACTAGTACCTGTAGGTTCCAAGGCATGG	*Spe* I
LC3-11	CCCAAGCTTACGGGTGATGGGCTCTTGGAG	*Hind*III
LC3-12	AAGCGGCCGCTCTAGATGAACATAGGCGTTTGGGTG	*Xba* I
LC3-13	ACGCGTCGACAAATGACTACTGAAGAACTCGCTT	*Sal* I
LC3-14	ACGCGTCGACAACTACTGTAGGTTCCAAGGCAT	*Sal* I
LC3-15	GCGTCGACATGACTACTGAAGAACTCGC	*Sal* I
LC3-16	GGGGTACCCTGTAGGTTCCAAGGCATGG	*Kpn* I
LIP1-1	GGAATTCATGGAGAGGGAAGTCGTGGTC	*EcoR* I
LIP1-2	CGGGATCCAATCTCCTCAATAAGAACCTTGG	*BamH* I
LIP1-3	GCGTCGACATGGAGAGGGAAGTCGTGGTC	*Sal* I
LIP1-4	GGACTAGTAATCTCCTCAATAAGAACCTTGG	*Spe* I
LIP1-5	GGCAGTCGTGGTCTCCGAGGACG	
LIP1-6	AAACCGTCCTCGGAGACCACGAC	
LIP1-7	GCCGTTCTCCCTCGCGGAAACCA	
LIP1-8	AAACTGGTTTCCGCGAGGGAGAA	
LIP1-9	GGAAACCAGGGTCATCTGCC	
LIP1-10	TGACTCATCTTCGCCTTCTGA	
IAA12-1	GCCGTACCTGAGGAAGATCG	
IAA12-2	TTGCCTTGGTGATCCTAGCC	
IAA12-3	CGGGATCCATGGAAGCCGCCGTGGGGTA	*BamH* I
IAA12-4	GGGGTACCGATCTTCATTTGCCTTGGTG	*Kpn* I
IAA12-5	CGGAATTCATGGAAGCCGCCGTGGGGTA	*EcoR* I
IAA12-6	GCGTCGACGATCTTCATTTGCCTTGGTGATCC	*Sal* I
IAA12-7	GCGTCGACATGGAAGCCGCCGTGGGGTA	*Sal* I
IAA12-8	GGACTAGTGATCTTCATTTGCCTTGGTGATCC	*Spe* I
IAA12-9	GATTTCTGAGGAGGATCTTCCCGGGGAAGCCGCCGTGGGGTACGC	*Sma* I
IAA12-10	AAGCAGGGCATGCCTGCAGGTCGACTTAGATCTTCATTTGCCTTG	*Sal* I
IAA12-11	ACTCACTATAGGGCGAATTCTCATAATTTATATTCCCAAT	*EcoR* I
IAA12-12	ATTCGCGAACGCGTGAGCTCTGCTTGATTTCTGGTGCACC	*Sac* I

IAA12-13	ACTCACTATAGGGCGAATTCGGTGCACCAGAAATCAAGCA	*EcoR* Ⅰ
IAA12-14	ATTCGCGAACGCGTGAGCTCTTCTCGCTTCCTCTCTAGCT	*Sac* Ⅰ
IAA12-15	GGGGTACCCACCGCAGTCATCTCGCTAT	*Kpn* Ⅰ
IAA12-16	TTGCGGCCGCTTCTCGCTTCCTCTCTAGCT	*Not* Ⅰ
IAA12-17	GATCAATAGGGCCGAAAGGG	
IAA12-18	GTTGATACAGAGACATAGTG	
IAA12-19	AATTTTCCGGGTTACCGGAT	
IAA12-20	CTTGATTATAAGGGTGTGGA	
IAA12-21	ATGGGACATAATAATTTAAA	
IAA12-22	GGGTTCATATTCTATTCCCC	
IAA12-23	CGGCGTGGCCCGGCGACCGG	
IAA12-24	GAGGAGTGCAAGTTTTATAG	
IAA12-25	TATAAAACTTGCACTCCTCC	
IAA12-26	TTCTCGCTTCCTCTCTAGCT	
IAA12-27	CGCCGCCGACAGCCTCATCA	
IAA12-28	TGCTCGTCGTCGTCGGCGGC	
GH3.2-1	GGGGAGAGGAAGCTAATGCC	
GH3.2-2	CTGCGCGTACATGGACTGGA	
GH3.2-3	GCCGCCCACCCCGCGAGTCC	
GH3.2-4	TGTATCTGTAAATTTATCGT	
GH3.2-5	TTGACTCACGAGAGGTTA	
GH3.2-6	CCGTGTCTTGACTTGTGC	
GH3.2-7	GTAAAACACGTTTGGCTCTT	
GH3.2-8	TAGTGGTACTTGTACGGCTG	
GH3.2-9	CGCGACGTGCATGGCGCG	
GH3.2-10	ATTAGTTGGCGTTGCTTGCC	
GH3.2-11	CCAAATACAGCCCGCACAAG	
GH3.2-12	ACTGGGACTGAGTGCTGGTA	
ARF17-1	GGCAAGGCAACGAAACAAGGGGAC	
ARF17-2	AAACGTCCCCTTGTTTCGTTGCCT	
ARF17-3	AGGAGGACCTGCATATGATGAGGCTTTCGTCGTCGTC	*Nde*Ⅰ
ARF17-4	GCAGGTCGACGGATCCGAATTCAACTGAGCCGACAGATGCAA	*BamH* Ⅰ
ARF17-5	CCCTCGAGATGAGGCTTTCGTCGTCGTC	*Xho* Ⅰ
ARF17-6	GGACTAGTACGAATTCAACTGAGCCGACAGATGCAA	*Spe* Ⅰ
ARF17-7	CGACTCTAGGAGCTCGGTACCCGGGATGAGGCTTTCGTCGTCGTC	*Sma* Ⅰ
ARF17-8	GAACATCGTATGGGTACATACTAGTGAATTCAACTGAGCCGACAGATGCAA	*Spe* Ⅰ
ARF17-9	GGATCAAGATGGGAACTCTG	
ARF17-10	TAGTCATCACAGCTGCTACC	
ARF2-S	CTGCTTCCAATTATTATTCCAGTAC	
ARF2-A	TCAACCCTTTTCTGTTGGAATGC	
ARF3-S	TCAAAGATGCGCACACTGTC	
ARF3-A	CATGCAGCTTCGTGTATCAG	
ARF11-S	CCACTGAGAGTTAAGCATCC	
ARF11-A	GCATTAGGATCAGAGAAGCC	
ARF15-S	ATGGACAGTTGGACAAGCAC	
ARF15-A	TCAGAATCATCAGTCCCTCC	
IAA6-S	AGCTTGGTCTTCCTGGTTTTAT	

IAA6-A	CACCTTTGCATTGGCATTATC	
IAA9-S	AAGAAAATGGCCAATGATGATCA	
IAA9-A	CCCATCACCATCCTCGTAGGT	
IAA24-S	GGCTTGTGCTCTTCGTTGCT	
IAA24-A	CCTCTTGGATTCAGAAACACTGAA	
IAA27-S	TCAGGGCACACCGCAACTA	
IAA27-A	TAGGGTACTCACGGCAGAAGAAA	
BAK1-S	GAGTTGATCTTGGGAATGCTGC	
BAK1-A	CACTAGGTATCGTTCCGCTTATGTT	
D2-S	CACTCCTTTTGGTGGTGGGC	
D2-A	GGTGGGGAAGTTGACGATGTG	
D3-S	AAGCCGGTTTATCCAATTCC	
D3-A	GCAC CAAGAATCGTCTGGAT	
D4-S	AGTCGCGTGCTGCCATTCTCGGAGTAATAG	
D4-A	AGCAAGCTCAGCAAGAGGTCCAGGATTTGC	
D14-S	GTGCTGTCGCATGGCTTC	
D14-A	GCAGGTCGTCG ACGTAGG	
D17-S	CATCTTCTCCGACGACCACG	
D17-A	CGTCTCCACGAACCTGCAT	
D27-S	TCTGGGCTAAAGAATGAAAAGGA	
D27-A	AGAGCTTGGGTCACAATCTCG	
LAZY1-S	CACAGGAAAGTCTACCCCGAG	
LAZY1-A	GGCTCATAAACACCAATCACATC	
LIC1-S	ATGCAGACAACAAATTTC	
LIC1-A	GCAACTAATATTGGACCGCTA	
TAC1-S	GAGATGGCTCTAAAGGTGTTC	
TAC1-A	CGTGCCAATTGCAAGTATACC	
